# The determinants of COVID-19 morbidity and mortality across countries

**DOI:** 10.1038/s41598-022-09783-9

**Published:** 2022-04-07

**Authors:** Dianna Chang, Xin Chang, Yu He, Kelvin Jui Keng Tan

**Affiliations:** 1 Singapore University of Social Sciences, Singapore, Singapore; 2 Nanyang Technological University, Singapore, Singapore; 3 Central University of Finance and Economics, Beijing, China; 4 The University of Queensland, Brisbane, Australia

**Keywords:** Health policy, Risk factors

## Abstract

We identify 21 predetermined country-level factors that explain marked variations in weekly COVID-19 morbidity and mortality across 91 countries between January and the end of 2020. Besides factors commonly associated with infectious diseases (e.g., population and tourism activities), we discover a list of country characteristics that shape COVID-19 outcomes. Among demographic–geographic factors, the male-to-female ratio, population density, and urbanization aggravate the severity of COVID-19, while education, temperature, and religious diversity mitigate the impact of the pandemic on morbidity and mortality. For the political-legal dimension, democracy and political corruption are aggravating factors. In contrast, female leadership, the strength of legal systems, and public trust in government significantly reduce infections and deaths. In terms of socio-economic aspects, GDP per capita, income inequality, and happiness (i.e., life satisfaction) lead to worse COVID-19 outcomes. Interestingly, technology advancement increases morbidity but reduces mortality. For healthcare factors, SARS (severe acute respiratory syndrome) experience and healthcare infrastructure help countries perform better in combating the pandemic.

## Introduction

The outbreak of COVID-19 has affected almost all countries and caused significant loss of life, health, and economic output. As of December 16, 2021, according to *Worldometer* (https://www.worldometers.info/coronavirus/), there have been 272.5 million confirmed cases of infection and 5.3 million deaths worldwide. Further, the International Monetary Fund reported a projected cumulative output loss from 2020 to 2025 of $22 trillion (https://www.imf.org/en/News/Articles/2021/01/28/tr012621-transcript-of-the-world-economic-outlook-update-press-briefing). During this global pandemic, many countries have adopted similar approaches to contain the spread of COVID-19; however, these countries exhibit drastic differences in morbidity and mortality, even among those with similar socio-economic conditions and political backgrounds. For example, Australia and New Zealand share similar economic and political environments. The population of Australia is approximately five times that of New Zealand (24.5 m vs 4.8 m). As of December 31, 2020 (the end of sample period), Australia had reported 28,405 infected cases and 909 deaths, about 13 times the 2162 cases and 36 times the 25 deaths reported by New Zealand, respectively. This study identifies and evaluates the primary country-specific factors that explain these cross-country variations in COVID-19 outcomes (i.e., morbidity and mortality).

In particular, we focus on country-level factors determined prior to the COVID-19 outbreak (e.g., GDP per capita, religious diversity, and democracy) instead of ex-post factors capturing a country’s responses to COVID-19 (e.g., contact tracing, lockdowns, and travel bans). We forgo ex-post response variables not because they lack importance; on the contrary, given the extraordinary speed and scope of the coronavirus crisis, government actions, measures, and policies have significantly affected national cases and death counts. For example, Balmford et al.^1^ use epidemiological models to analyze COVID-19 cases and deaths across all OECD countries as of June 9, 2020, and show that eight countries (Belgium, China, Denmark, Germany, Italy, Korea, the U.K., and the U.S.) might have lost more than half a million lives by delaying lockdowns for a week. Nevertheless, we exclude response variables because they are highly endogenous. A response measure (e.g., lockdowns and vaccination) may enable countries to lower the number of COVID-19 cases and deaths. Conversely, countries with greater COVID-19 cases and deaths are more likely to adopt such a measure. Thus, the relation between a response variable and COVID-19 outcomes is often intertwined, making it difficult for researchers to disentangle the confounding effects.

In contrast, predetermined country characteristics are less subject to endogeneity concerns and confounding issues because they are beyond policymakers’ control within a relatively short period. For instance, a country cannot quickly adjust its population’s education level during its battle against the coronavirus pandemic. In econometric terms, the current and lagged values of our predetermined country-level variables are uncorrelated with the error terms of the current period in the regression analyses, thereby mitigating endogeneity concerns. Further, our approach essentially assumes that predetermined country characteristics affect COVID-19 outcomes both directly and indirectly. For example, the population density of a country should affect its COVID-19 outcomes directly since relatively high population density may foster the spread of infectious diseases^2^. On the other hand, population density may also affect COVID-19 outcomes indirectly via shaping governments’ response measures (e.g., lockdowns), which can be more challenging for those living in rural areas to cope with. Our analyses combine both the direct and indirect effects of predetermined country characteristics on COVID-19 cases and deaths.

Our sample initially consists of 99 countries, for which 20 country-level determinants are available from various databases and sources. The sample reduces to 91 countries when we include an additional determinant to account for people’s trust in government and the number of COVID-19 tests as a control variable, data for which are unavailable for eight countries. The inclusion of the number of COVID-19 tests aims to account for the mechanical relation between COVID testing and confirmed cases. We group the determinants into four categories: demographic-geographic, political-legal, socio-economic, and health factors. We gather the weekly numbers of COVID cases and deaths reported between the start of the coronavirus outbreak (January 22, 2020) and December 31, 2020, from the website of the Johns Hopkins Coronavirus Resource Center. We stop the sample at the end of 2020 to ensure that our analyses are unaffected by the COVID-19 vaccination, which has been rolled out since January 2021 in many countries (https://ourworldindata.org/covid-vaccinations). While a few countries (e.g., Israel, the U.S., Bahrain, and Germany) started COVID-19 vaccinations in December 2020, vaccination coverage in the early days of the roll-out was not large enough to significantly change the COVID-19 trends up to December 31, 2020.

As our sample includes multiple countries across time, we employ Fama and MacBeth’s two-step approach^3^ to ensure that we compare all countries at the same point in time. Specifically, we run a cross-sectional regression across all countries each week and generate a time series of the coefficient estimates. We then calculate the mean values of the weekly coefficient estimates and assess their statistical significance using *t*-tests. This approach also allows us to keep track of how the coefficients of country-level determinants vary over time.

Our regression analysis reveals that the 21 factors (excluding the number of tests, which is not a predetermined factor) are robustly significant in our tests and collectively explain 78% (72%) of the cross-country variations in the number of confirmed infections (deaths). Of the 21 determinants, 12 are aggravating factors that significantly drive up COVID infection cases, including five demographic–geographic (population, population density, the median age of the population, the male-to-female ratio, and urbanization), two political-legal (democracy and corruption), and five socio-economic (GDP, technology, income inequality, happiness, and tourism) factors. These factors are also positively associated with the number of deaths, except technology, which is negatively related to COVID-19 mortality. This finding suggests that technology enables governments to quickly identify actual and potential COVID-19 infections through, for example, digital contact tracing, thereby resulting in more confirmed cases. Meanwhile, technology also facilitates early screening and timely treatment, leading to fewer deaths.

In addition, we identify nine mitigating factors that reduce infection cases: three demographic-geographic (temperature, education, and religious diversity), four political-legal (media freedom, female leadership, trust in government, and law), and two healthcare (SARS experience and healthcare infrastructure) factors. These factors are also negatively associated with the number of deaths, except media freedom. All results discussed above are robust to including the number of COVID-19 tests as a control variable.

We then examine how the relation between a country-level determinant and COVID-19 outcomes varies over time by plotting the weekly coefficients. We find that most relations are highly persistent over time. In addition, we perform dominance analysis to evaluate the relative statistical importance of the factors in explaining cross-country differences in COVID-19 morbidity and mortality. The results show that the following top five determinants combined explain approximately 62.5% of cross-country variations in the number of confirmed COVID-19 cases explained by all determinants: (1) population, (2) international tourism activity, (3) SARS experience, (4) happiness, and (5) technology. Finally, we evaluate the margin effect of a one-standard-deviation change in a determinant on pandemic outcomes, and find that population, temperature, median age of the population, happiness, and corruption are the five determinants with the most substantial marginal effects.

COVID-19 has had devastating consequences for public health and economies throughout the world. Our country-level determinants of COVID-19 outcomes highlight the importance of prevention rather than treatment in healthcare, as prevention can be more effective than a cure^4^. Our findings suggest that factors seemingly unrelated to healthcare (e.g., female leadership) can play essential roles in pandemic prevention, thereby shaping pandemic control outcomes. Although the documented relations between the predetermined factors and the COVID-19 outcomes reflect correlation rather than causation, our findings shed light on public policies that help develop preventive resources so that countries can better prepare for future health crises. Moreover, to the best of our knowledge, our study examines the most extensive set of determinants across a large number of countries for cross-country comparisons of COVID-19 outcomes. The determinants that we identify can be used as important controls by future empirical work on pandemic-induced morbidity and mortality across nations.

## Data and variables

### Sample

Our sample period runs from January 22 to December 31, 2020. We begin the sample period on January 22—the date of the first confirmed case of COVID-19 in China. We collect COVID-19 related information from the Coronavirus Resource Center of Johns Hopkins University (https://coronavirus.jhu.edu/map.html). Specifically, we use two measures as our dependent variables: the weekly number of confirmed COVID cases and the weekly number of COVID-19 deaths. The country-level determinants are collected from several databases and sources, detailed in Supplementary Information A. We require that all countries have no missing values for these variables. Our final sample contains 99 countries and regions (listed in Supplementary Information B). Specifically, our sample consists of 18 African, 24 Asian, 34 European, 10 North American, three Oceanian, and 10 South American countries/regions. These countries/regions collectively account for 93.5% (untabulated) of the 2020 global GDP. According to the World Bank’s World Development Indicators (WDI), 41 countries in our sample are high-income, 51 are middle-income, and seven are low-income countries/regions.

### Variable descriptions

We construct the two main dependent variables by taking the natural logarithm of one plus the weekly confirmed cases (*Ln*(1 + *Confirmed*)) and deaths (*Ln*(1 + *Death*)), respectively. We log-transform the two variables to reduce right skewness and achieve normality. Further, SARS-CoV-2, the virus that causes COVID-19, tends to spreads exponentially rather than arithmetically, especially in the initial outbreak phases. Thus, log transformation is a natural way to track the spread of the virus. In our baseline regressions, we do not scale the dependent variables using population size. While numbers adjusted for population are good at capturing how much relative strain a country is under, they are not suitable for tracking the extent or the state of a country’s outbreak^5^. Specifically, Nace^5^ uses the following scenario to illustrate the problem of scaling coronavirus cases by population: with 10,000 coronavirus cases in a country of 300 million people and another country of 30 million people, the virus will spread at the same pace, while holding all other factors equal. Dietz and Heesterbeek^6^ also show that the growth of infectious viruses is relatively independent of the total population size. Therefore, the raw numbers of infected cases reveal more about the coronavirus situation of a country. Taken together, we employ the raw numbers of infected cases and deaths as dependent variables, and include population size as an explanatory (control) variable in our regression analyses. As a robustness check, we also define the dependent variables as the natural logarithm of one plus the number of confirmed cases (deaths) per million people and obtain qualitatively similar results (tabulated in Supplementary Information C).

To explain the cross-country differences in weekly confirmed cases (deaths), we consider four groups of country-level variables: (1) demographic–geographic, (2) political-legal, (3) socio-economic, and (4) healthcare. First, to capture the demographic and geographic aspects of a country, we include the following eight proxies: the natural logarithm of the total population in thousands (*population*), population density (*population density*), the median age of the population (*age*), the number of males per 100 females in the population (*male*), the proportion of people living in urban areas (*urbanization*), the weekly average temperature in Celsius from January 22 to the end of each week (*temperature*), the population’s education level (*education*), and the religious fractionalization index (*Religious Diversity*) as a proxy for religious heterogeneity.

Regarding the political and legal aspects, we include six proxies: *democracy*, which measures whether there are free and fair elections in a country and how responsive the government is to its people; a corruption index (*corruption*); the degree of media freedom (*media freedom*), measured using a world press freedom index, constructed by pooling the responses of experts to a questionnaire devised by Reporters Without Borders (RSF); an indicator variable (*female leader*) that equals one if the highest political position in a country is a female and zero otherwise; the percentage of people who trust their national government (*trust government*); and the strength and impartiality of the legal system (*law*). For social and economic aspects, we include five proxies: GDP per capita (*GDP*) adjusted for purchasing power parity (PPP), the Gini coefficient as a measure of inequality (*inequality*), the number of arrivals of non-resident tourists at national borders as a proxy for tourism activities (*tourism*), technology (*technology*), which aggregates three essential technology proxies covering investment in emerging technology, 4G mobile network coverage, and the use of virtual social networks, and the happiness score (*happiness*) published by the World Happiness Report as a proxy for self-reported life satisfaction. For the healthcare dimension, we include the number of SARS cases from November 1, 2002, to July 31, 2003, to quantify each country’s SARS experience (*SARS*), and the number of hospital beds that are regularly maintained and staffed and immediately available for the care of admitted patients, as a measure of public health resources (*hospital beds*). Finally, we include the number of COVID-19 tests (per hundred people) performed in a country (*N_Tests*) as a control variable.

### Descriptive statistics

**Figure 1:**
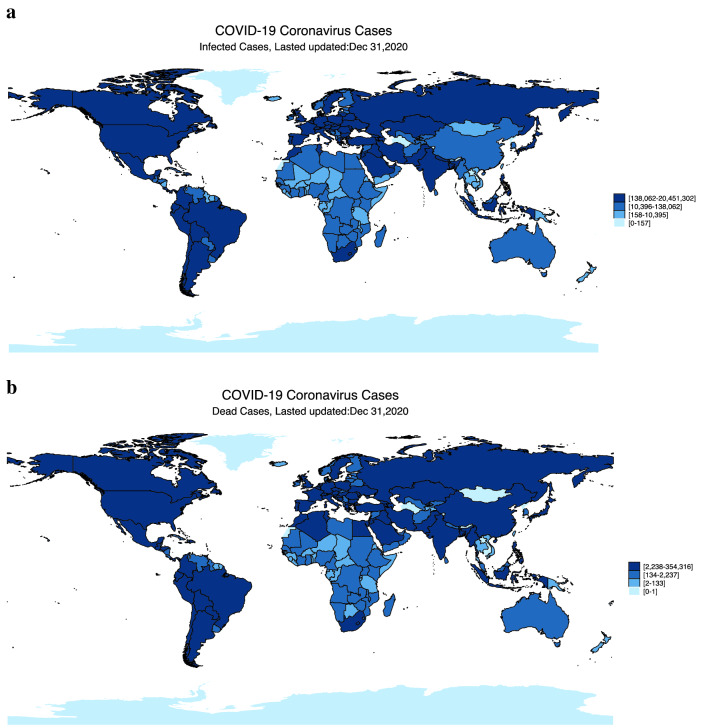
The number of confirmed COVID-19 cases and deaths across the globe. **(a)** Shows the distribution of the number of confirmed COVID-19 cases across the globe based on the four quintiles of total confirmed infected cases as of December 31, 2020: (1) 0 to 157, (2) 158 to 10,395, (3) 10,396 to 138,062, and (4) 138,062 to 20,451,302. The darker the color, the higher the number of confirmed COVID-19 cases in the country. For more details, please see Supplementary Information D. **(b)** Shows the distribution of the number of deaths across the globe based on the four quintiles of total deaths as of December 31, 2020: (1) 0 to 1, (2) 2 to 133, (3) 134 to 2237, and (4) 2238 to 354,316. The darker the color, the higher the number of COVID-19 deaths in that country.

Figure 1a,b present the distributions of confirmed COVID-19 cases and deaths across the globe, respectively. We group countries into four quartiles based on the severity of COVID outcomes. Notably, the two graphs show that the U.S., India, Brazil, Russia, and France have the greatest cumulative number of confirmed cases by the end of 2020; the five countries with the highest number of deaths in that period are the U.S., Brazil, India, Mexico, and Italy.

Table 1 shows the summary statistics for the dependent and explanatory variables used in the COVID-19 severity regressions. The average age of the population in our sample is 32.8 years old, the average number of years of schooling is 14.4, and about 66.5% of people live in urban areas. The average GDP per capita is $26,850, about 14% of the countries in our sample are led by female leaders, and about 30% of people received COVID-19 tests. The countries in our sample exhibit significant political and cultural diversities. For example, standard deviations of religious diversity, the level of democracy, trust in government, and inequality are 2.124, 1.382, 17.631, and 7.395, respectively.

**Table 1:** Descriptive statistics.

	N	Mean	Standard deviation	25th percentile	Median	75th percentile
**Dependent variables**						
*Confirmed*	4851	211,775	975,979	884	10,929	86,102
*Death*	4851	6364	23,111	11	229	2261
*Ln*(*1* + *Confirmed*)	4851	8.473	3.991	6.786	9.299	11.363
*Ln*(1 + *Death*)	4851	5.140	3.352	2.485	5.438	7.724
**Demographic-geographic factors**						
*Population*	99	9.758	1.571	8.665	9.754	10.813
*Population density*	99	0.303	1.085	0.032	0.094	0.206
*Age*	99	32.795	8.854	27.100	32.800	41.400
*Male*	99	97.844	6.145	94.887	98.083	100.244
*Urbanization*	99	66.534	20.078	54.084	69.051	82.459
*Temperature*	4851	14.477	10.203	7.600	15.346	23.624
*Education*	99	14.439	2.772	12.600	14.700	16.300
*Religious diversity*	99	3.298	2.124	1.400	3.100	5.300
**Political-legal factors**						
*Democracy*	99	4.463	1.382	3.500	4.583	6.000
*Corruption*	99	− 2.945	1.269	− 4.000	− 2.500	− 2.000
*Media freedom*	99	− 31.161	13.763	− 40.950	− 30.160	− 22.670
*Female leader*	99	0.141	0.348	0.000	0.000	0.000
*Trust government*	93	52.821	17.631	40.300	51.434	64.537
*Law*	99	3.771	1.281	2.708	3.500	5.000
**Socio-economic factors**						
*GDP*	99	26.850	22.235	8.762	20.611	40.112
*Inequality*	99	37.807	7.395	32.225	36.560	42.812
*Tourism*	99	12.407	18.009	1.781	4.425	15.347
*Technology*	99	59.446	19.200	48.556	62.275	75.596
*Happiness*	99	5.752	1.070	4.981	5.890	6.399
**Healthcare factors**						
*SARS*	99	0.595	1.481	0.000	0.000	0.000
*Hospital beds*	99	3.327	2.525	1.400	2.800	4.700
**Control variable**						
*N_Tests*	97	29.883	38.787	4.662	18.395	42.173

Table shows the summary statistics for the dependent variables and cross-country determinants in the COVID-19 regressions. The sample period is from January 22, 2020 to December 31, 2020. Details of all variable definitions and data sources are in Supplementary Information A. *N_Tests* is the number of COVID-19 tests (per hundred people) performed in a country.

## Empirical results

We conduct Fama and MacBeth regressions^3^ to test the cross-country determinants of the morbidity and mortality rates of COVID-19. Specifically, we estimate a cross-country regression for each week, and then calculate the average values of the weekly estimates. The primary benefit of using this model is to correct standard errors for cross-sectional correlations^7^. The model essentially explores cross-country variations by comparing countries in the same calendar week and allows the coefficients of country-level determinants to vary over time. We present the baseline regression results in Table 2. Specifically, we estimate the following baseline model using Fama and MacBeth’s approach^3^:

1 $$Ln\left( {{1} + Y_{i,t} } \right) = \, \alpha \, + \, \beta X_{i,t} + \, \varepsilon_{i,t} ,$$

where *Y*_*i,t*_ represents the number of confirmed cases or deaths in country *i* in week *t*. *X* is the set of explanatory variables defined in “Variable descriptions” section and tabulated in Supplementary Information A.

**Table 2:** Fama–MacBeth regressions for confirmed cases and deaths.

Dependent variables	(1)	(2)	(3)	(4)
	*Ln*(1 + *Confirmed*)		*Ln*(1 + *Death*)	
*Population*	0.935*** (18.7)	0.963*** (18.6)	1.030*** (14.4)	1.052*** (14.8)
*Population density*	0.314*** (6.3)	0.322*** (8.7)	0.053 (1.4)	0.065** (2.7)
*Age*	0.049*** (11.1)	0.054*** (11.2)	0.087*** (21.4)	0.078*** (16.6)
*Male*	0.027*** (12.8)	0.023*** (5.8)	0.025*** (14.3)	0.032*** (8.9)
*Urbanization*	0.011*** (20.5)	0.012*** (17.4)	0.017*** (23.1)	0.016*** (17.9)
*Temperature*	− 0.065*** (− 8.6)	− 0.062*** (− 8.9)	− 0.064*** (− 10.3)	− 0.072*** (− 11.5)
*Education*	− 0.052*** (− 11.5)	− 0.077*** (− 15.6)	− 0.072*** (− 15.4)	− 0.089*** (− 12.0)
*Religious diversity*	− 0.145*** (− 11.7)	− 0.133*** (− 12.8)	− 0.202*** (− 11.8)	− 0.194*** (− 13.4)
*Democracy*	0.150*** (8.6)	0.108*** (6.1)	0.177*** (12.8)	0.174*** (13.9)
*Corruption*	0.292*** (23.6)	0.266*** (20.8)	0.199*** (15.4)	0.153*** (14.3)
*Media freedom*	− 0.001 (− 0.9)	− 0.004*** (− 3.2)	0.007*** (3.4)	0.004*** (3.5)
*Female leader*	− 0.254*** (− 5.3)	− 0.105*** (− 3.7)	− 0.380*** (− 8.0)	− 0.399*** (− 12.0)
*Trust government*		− 0.006*** (− 6.2)		− 0.007*** (− 7.1)
*Law*	− 0.132*** (− 3.5)	− 0.001 (− 0.0)	− 0.218*** (− 8.8)	− 0.138*** (− 6.5)
*GDP*	0.016*** (16.7)	0.014*** (13.7)	0.017*** (12.0)	0.021*** (14.2)
*Inequality*	0.012*** (3.9)	0.018*** (9.1)	0.015*** (5.5)	0.017*** (10.8)
*Tourism*	0.009*** (10.9)	0.007*** (6.1)	0.014*** (8.9)	0.013*** (7.3)
*Technology*	0.014*** (9.1)	0.012*** (7.0)	− 0.002** (− 2.3)	− 0.002* (− 1.7)
*Happiness*	0.359*** (10.0)	0.328*** (8.6)	0.351*** (8.8)	0.362*** (8.8)
*SARS*	− 0.246*** (− 4.1)	− 0.211*** (− 5.1)	− 0.231*** (− 5.1)	− 0.332*** (− 11.4)
*Hospital beds*	− 0.110*** (− 7.7)	− 0.117*** (− 7.7)	− 0.178*** (− 13.4)	− 0.188*** (− 13.5)
*N_Tests*		0.002*** (3.6)		− 0.002*** (− 6.7)
*Constant*	− 6.102*** (− 13.9)	− 5.959*** (− 8.3)	− 10.042*** (− 16.5)	− 10.472*** (− 16.1)
N	4851	4459	4,851	4459
R^2^	0.790	0.790	0.750	0.721

Table presents the Fama–MacBeth regression results of confirmed COVID-19 cases and deaths. The dependent variable in columns (1) and (2) is the natural logarithm of one plus the confirmed cases, while the dependent variable in columns (3) and (4) is the natural logarithm of one plus the number of deaths. The definitions of all country-level variables are described in Supplementary Information A. Columns (2) and (4) further control for the number of COVID-19 tests (*N_Tests*) conducted and *Trust Government*. The *t*-statistics are reported in parentheses, and ***, **, and * indicate significance at the 1%, 5%, and 10% levels, respectively.

In Table 2, the dependent variable in columns (1) and (2) is the number of confirmed cases (*Ln*(1 + *Confirmed*)), while the dependent variable in columns (3) and (4) is the number of deaths (*Ln*(1 + *Death*)). In contrast to columns (1) and (3), columns (2) and (4) further control for *trust government* and the number of COVID-19 tests conducted (*N_Tests*), which are unavailable for eight countries. Specifically, the proportion of people who trust the national government is missing for six countries. The number of cases tested is missing for two countries. As a result, we lose about 8% of the observations with the inclusion of the two extra explanatory variables. In untabulated analyses, we find that the mean variance inflation factor is less than 4.2, suggesting that multicollinearity is not a major issue in our setting^8^.

Based on the signs of the estimated coefficients, we categorize country-level determinants into two groups: aggravating factors, which have positive signs in the regressions, and mitigating factors, which have negative coefficients in the regressions. We discuss the two groups of factors in “Aggravating factors” and “Mitigating factors” sections, respectively.

### Aggravating factors

#### Population

Table 2 shows that a country’s population is positively and significantly correlated with total infection cases and deaths. This result is unsurprising because countries with larger populations (e.g., China, the U.S., and India) generally have more infections and deaths reported in a given week. To quantify the effect of the change in *population* on the number of confirmed cases in column (1), if we increase *population* by one standard deviation (1.571), the increase in the number of confirmed cases (*Confirmed*) from its mean value (211,775) is equal to 0.935 × (1 + 211,775) × 1.571 = 311,075, which amounts to 147% of the mean value of *confirmed*. Because *d*[*Ln*(1 + *confirmed*)]/*d*(*population*) = [1/(1 + *confirmed*)] × [*d*(*confirmed*) /*d*(*population*)], *d*(*confirmed*) = [*d*[*Ln*(1 + *confirmed*)]/*d*(*population*)] × (1 + *confirmed*) × *d*(*Population*). Similarly, a one-standard-deviation increase in *population* is associated with a 162% increase in the number of deaths (*Death*) from its mean value.

**Figure 2:**
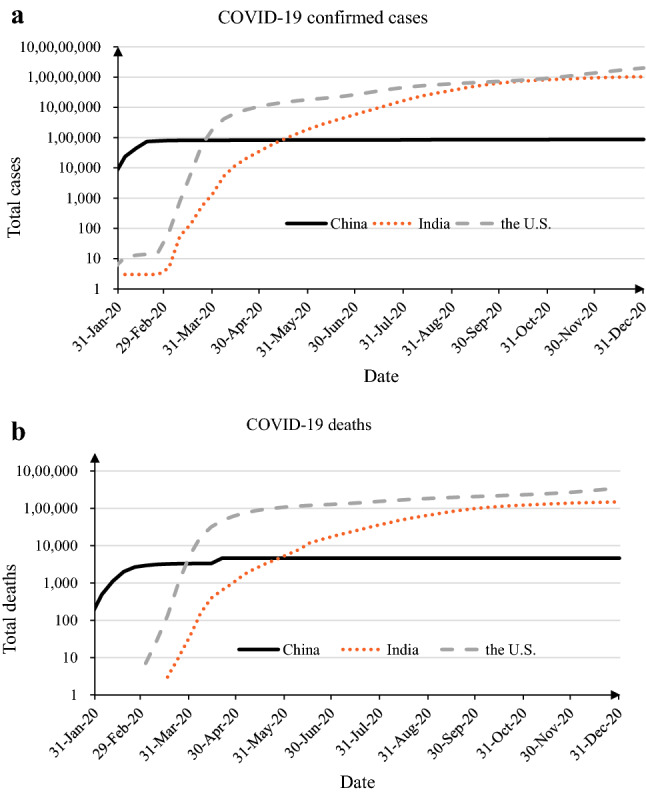
Cumulative COVID-19 cases and deaths in China, India, and the U.S. **(a)** Shows the cumulative confirmed COVID-19 cases in China, India, and the U.S. between January 22, 2020 and December 31, 2020. The y-axis is on a logarithmic scale. **(b)** Shows the cumulative COVID-19 deaths in China, India, and the U.S. between January 22, 2020 and December 31, 2020. The y-axis is on a logarithmic scale.

In Fig. 2, we plot the cumulative number of infections and deaths (with the y-axis on a logarithmic scale) for the three most populous countries (India, China, and the U.S.). These exhibit different trends over time: China reported the largest total coronavirus cases at the beginning of the pandemic, whereas the U.S. (India) reported drastic surges in infections and deaths from March (April) 2020 onwards. Thus, China contributes more to the positive effect of population on COVID morbidity and mortality in the early stages of the pandemic, while other populous countries (e.g., India and the U.S.) drive the population effect in more recent periods.

#### Population density

The results in Table 2 show that population density has a significantly positive effect on confirmed infections and deaths, suggesting that the Coronavirus causes more infections and spreads faster in more densely populated regions. The effect of population density on COVID-19 outcomes is consistent with Dietz and Heesterbeek^6^ and Nace^5^, who document population density as playing an important role in driving how fast a virus spreads. Our finding also supports the physical distancing rule recommended by the World Health Organization, which requires people to keep a distance of at least 1 m from one another and avoid spending time in crowded places or in groups (https://www.who.int/westernpacific/emergencies/covid-19/information/physical-distancing). Using the coefficients of *population density* in columns (2) and (4), we can infer that a one-standard-deviation (i.e., 1.085) increase in *population density* is associated with an increase of 34.94% (7.05%) in *confirmed* (*death*) from its mean value. The coefficient of *population density* is positive but statistically insignificant in column (3) (*t*-statistic = 1.4) where the dependent variable is *Ln*(1 + *death*). Thus, we discuss the marginal effects of *population density* using the coefficients in columns (2) and (4), where we include all explanatory variables.

#### Age

The positive and significant coefficients of *Age* indicate that, *ceteris paribus*, COVID-19 infection and death rates are higher for countries with older populations. These results are consistent with Dowd et al.^9^, who document for Italy that the chances of infection and death are significantly higher for the elderly than for younger people. Similarly, Boehmer et al.^10^ and the U.S. Centers for Disease Control and Prevention (CDC) document consistent findings using U.S. data. For example, the CDC reports that, relative to the death rate of the control group aged 18–29 years, those of people in the age groups of 75–84 and 85 + years are about 220 times and 630 times higher, respectively (https://www.cdc.gov/coronavirus/2019-ncov/covid-data/investigations-discovery/hospitalization-death-by-age.html). We complement these country-specific studies by showing that *Age* consistently helps explain the variations in infections and deaths across a large number of countries. Using the coefficients of *Age* in columns (1) and (3), we can infer that a one-standard-deviation (i.e., 8.854) increase in *age* is associated with an increase of 43.4% (77.0%) in *confirmed* (*death*) from its mean value.

#### Male

The positive coefficients of the gender ratio, defined as the number of males per 100 females, suggest that males are more prone to contracting COVID-19 and subsequent death than females. A possible explanation is that males participate more in the labor market, which requires them to interact with other people, thereby increasing their likelihood of contracting the virus. In addition, Bwire^11^ points out that sex-based immunological differences can also cause higher morbidity and mortality in males; in particular, males have higher expression of angiotensin-converting enzyme-2 (ACE 2) than females, which is the receptor for both the SARS-coronavirus (SARS-CoV) and SARS-CoV-2. Moreover, differences in gender behavior or lifestyles can also play an important role. For instance, compared with women, men engage in higher levels of smoking and drinking, display less responsible attitudes/behaviors in undertaking preventive measures (e.g., frequent handwashing and wearing face masks), and are more likely to violate coronavirus policies (e.g., stay-at-home orders)^12^, thereby making themselves more vulnerable to COVID-19.

#### Urbanization

*Urbanization* measures the proportion of the total population living in urban areas as defined by national statistical offices. The ex-ante prediction regarding the effect of *Urbanization* on COVID-19 outcomes is ambiguous. On the one hand, people living in cities with high population densities are more likely to come into close contact with others. Hence, a higher degree of urbanization may aggravate the spread of COVID-19. On the other hand, rural areas may have less awareness of the pandemic and lack high-quality healthcare facilities, which may result in a higher chance of infection and death. Thus, the net effect of *Urbanization* on COVID-19 outcomes should be determined by the tension between these two opposing forces, and is best determined empirically. Our results in Table 2 show that *Urbanization* is significantly and positively correlated with both infected cases and deaths. This finding confirms that the greater levels of economic and social activity in urban areas facilitate congregation and render city dwellers more prone to infection than rural inhabitants. This effect dominates the plausible impact of low awareness of COVID-19 and poor medical facilities in rural areas on the spread of COVID-19. As such, our results are consistent with Rocklöv and Sjödin^2^, who document that contagious diseases spread more rapidly in cities than in low-density rural settings.

#### Democracy

*Democracy* measures free and fair elections and the degree of government interventions in a country. A priori, the relation between the level of democracy and a country’s success in combating the coronavirus pandemic is not immediately clear. Theoretically, democracy should boost public health because, in a democratic country, people can vote out a government that fails to improve healthcare quality and safety in favor of one that promises such improvements. In contrast, autocratic governments with poorly performing healthcare systems face no such checks. Further, democratic countries usually adopt broad-based policies and allocate resources more evenly, whereas authoritarian governments may favor certain groups. Consistent with the beneficial role of democracy in public health, Bollyky et al.^13^ document that the democratic experience of a country positively affects the health of its citizens. Similarly, using data on all epidemics from 1960 to February 2020, an analysis by *The Economist* shows that, on average, democratic countries experience lower mortality rates for epidemic diseases than their non-democratic counterparts (https://www.economist.com/graphic-detail/2020/02/18/diseases-like-covid-19-are-deadlier-in-non-democracies). The analysis further points out that “authoritarian regimes, although able to co-ordinate massive construction projects, may be poorly suited to matters that require the free flow of information and open dialogue between citizens and rulers”. Further, Chang et al.^14^ suggest that eight of the 10 best-performing countries in the battle against COVID-19, including New Zealand, South Korea, and Scandinavian countries (e.g., Finland, Norway, and Denmark), are democratic.

Conversely, democracies’ pandemic-fighting ability may be hampered by their inherent inefficiency and political division. During an interview with the BBC News on December 14, 2020, Dr. Anthony Fauci, the Director of the National Institute of Allergy and Infectious Diseases, stated: “[in the U.S.] each state has a considerable degree of independence of doing things the way they want to do … but when you are dealing with pandemic virus that knows no border or boundary between states, when you do things in a different way, it does not lead to an optimal response. Unfortunately, that is what happened.” (https://www.bbc.co.uk/programmes/m000qpcc) Cheibub et al.^15^ find that unlike autocratic countries, democratic countries have been slower to implement lockdown measures, which are often viewed as contrary to liberal rights. As the speed of government responses and interventions is critical for epidemic prevention and control, an authoritarian government may be able to effectively mitigate and contain the pandemic by making decisive decisions and mobilizing all its private and public institutions, as well as its entire population. In addition, democracy inevitably engenders individualism^16^, which has been shown by Huang et al.^17^ to aggravate the severity of COVID-19 by reducing the effectiveness of social distancing and mobility restriction policies.

In summary, recent studies provide contrasting predictions about the relation between democracy and a country’s performance against COVID-19. Thus, it is ultimately an empirical question to assess such relation during this pandemic. Our results in Table 2 are consistent with the view that, *ceteris paribus*, democratic countries have disadvantages in combating coronavirus relative to autocratic governments. The coefficients on *democracy* are positive and statistically significant in all regressions, indicating that, on average, more democratic countries experience higher rates of morbidity and mortality than less democratic countries. Using the coefficient in columns (1) and (3), we can infer that a one-standard-deviation (i.e., 1.382) increase in *democracy* is associated with an increase of 20.73% (24.47%) in *confirmed* (*death*) from its mean value.

We emphasize that our findings are context-specific and by no means serve as evidence of autocracies triumphing over democracies. While democracies have compelling advantages over autocracies in improving a country’s growth, development, human rights, life expectancy, and healthcare systems (e.g.^13,18–21^), the ongoing global pandemic may have exposed the weakness of democracies in combating highly contagious viruses, such as SARS-CoV-2. Instead of ensuring that more voices are heard during the pandemic, perhaps fast, coordinated, and collective actions are required to contain the spread of COVID-19. Having said that, it is immensely challenging for countries and governments to achieve the fine balance between control of the coronavirus outbreak and the effects of such control measures on the economy, income, education, and psychological wellbeing. The long-term and big-picture effects of both COVID-19 and related control measures are still unclear; autocratic countries and their residents may bear costs elsewhere, despite achieving short-term successes in the battle against COVID-19.

#### Corruption

*Corruption* measures the level of political corruption within a country’s political system, a factor that reduces government and business operational efficiency. As expected, the coefficients of *corruption* reveal that political corruption positively correlates with the severity of COVID-19. This finding suggests that political corruption contributes to the inefficiencies of government interventions to control the pandemic. Given the importance of being able to speedily mobilize all available resources to control and combat highly contagious diseases, curbing corruption during the COVID-19 pandemic is perhaps more important than ever.

#### GDP

Consistent with Feng et al.^22^, we document a positive relation between a country’s GDP per capita and the severity of COVID-19, suggesting that the pandemic results in more confirmed cases and deaths in developed countries than in developing countries. This result seems counter-intuitive, as one might expect that wealthier nations would have more resources to limit the spread of a pandemic^1^. However, we observe that many countries with a high GDP per capita (e.g., the U.S., Switzerland, the U.K., and France) also have high levels of COVID-19 morbidity and mortality. Schellekens and Sourrouille^23^ document that the coronavirus death toll has been concentrated in high-income countries. There are several potential explanations for these seemingly surprising results.

First, higher GDP per capita may imply greater levels of economic activity and international trade, leading to more frequent interactions among people and hence COVID-19 transmissions. Thus, our findings suggest that economic activity could act as a major mechanism through which an epidemic spreads. Second, Abdalla et al.^24^ find that advancement in healthcare or medicine has no direct relation with disease prevention. Wealthy countries, such as the U.S., may have outperformed other countries in many dimensions of healthcare but have overlooked investments in promoting population health and disease prevention. Third, developed countries are usually more liberal, so their governments may encounter greater resistance from citizens when implementing restrictive measures (e.g., lockdowns). Fourth, developing countries on average have younger populations, and are thereby less vulnerable to coronaviruses than developed countries, all else constant^23^. Last, inequality in data quality may play a role in driving the differences in reported numbers between developing and developed countries. We discuss this issue in “Discussion and conclusions” section, although Schellekens and Sourrouille^23^ point out that inequality in data quality is unlikely to be a first-order reason for the observed discrepancies between wealthy and poor countries.

#### Technology

Our technology measure focuses on three technology aspects that are highly relevant for infection prevention and control of pandemic-prone acute respiratory diseases: (1) investment in emerging technology, (2) 4G mobile network coverage, and (3) use of the virtual social network. In particular, (1) and (2) are related to technologies that enable advanced monitoring, surveillance, detection, and prevention through control measures such as contact tracing^25^, while (2) and (3) facilitate information dissemination, allow expeditious and coordinated data access and sharing, and promote collective actions and informed decision-making (e.g.^26,27^). Despite concerns over inaccurate information circulated on social media sites, Cinelli et al.^27^ report that the vast majority of information on mainstream social media sites is reliable (around 95% for Reddit, 93% for YouTube, and 89% for Twitter). We use the average value of these three aspects to capture the level of technological innovation.

Table 2 shows that *technology* is significantly and positively (negatively) related to the number of COVID-19 confirmed cases (deaths). These results are particularly interesting. The positive relation between *technology* and confirmed cases reflects the fact that technology can facilitate infectious disease control (e.g., population surveillance, identification of infected cases, contact tracing, and evaluation of interventions) and ensure that infected people and their close contacts are identified, traced, isolated, tested, and eventually confirmed^26^. On the other hand, early and rapid technology-driven identification of COVID-19 cases enables infected people to obtain timely treatment, which prevents severe consequences such as death, implying a negative relation between *technology* and the number of deaths.

#### Inequality

Our results show that income inequality, which is measured using the Gini coefficient, is significantly and positively correlated with confirmed cases and deaths. As earlier research (e.g.^28,29^) suggests, the poor, who are typically low-skilled workers, have a higher likelihood of being exposed to infectious diseases because they often need to be physically present at their workplaces (e.g., factories and construction sites) and use public transport. Further, the poor may prioritize daily necessities for living over their health. As such, low-income populations are more exposed to health risks and infectious diseases.

#### Tourism

**Figure 3:**
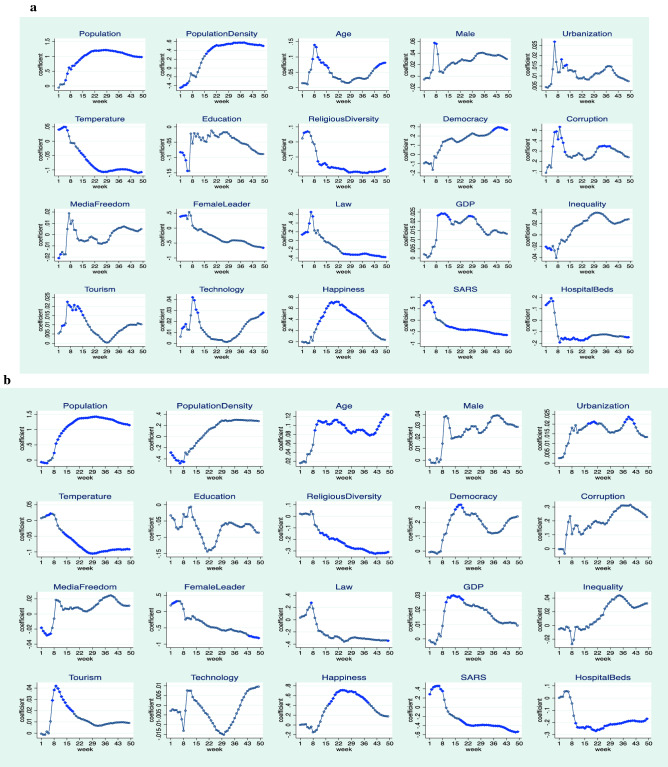
The coefficients of country-level determinants by week. **(a)** Shows the weekly coefficients of country-level determinants over time, with the dependent variable being *Ln*(1 + *Confirmed*). The blue solid points represent coefficients significant at the 10% level, while the hollow points represent statistically insignificant coefficients. **(b)** shows the weekly coefficients of country-level determinants over time, with the dependent variable being *Ln*(1 + *Death*). The blue solid points represent coefficients significant at the 10% level, while the hollow points represent statistically insignificant coefficients.

We find that *tourism*, measured by the number of non-resident tourist arrivals, is positively associated with the number of confirmed cases and deaths, indicating that mobility between countries facilitates virus transmissions. This result also highlights the importance of travel restrictions and border controls in limiting the spread of COVID-19 (e.g.^30^). The time-varying coefficients of *tourism* in Fig. 3 indicate that the aggravating effect of tourism on COVID-19 severity mainly took place in the first half of 2020. This is consistent with the findings of the Pew Research Center, which reveal that, as of March 31, 2020, 142 countries had complete (64 countries) or partial (78 countries) border closures put in place because of the COVID-19 outbreak (https://www.pewresearch.org/?p=361112).

#### Happiness

The coefficients of *happiness* indicate that the higher the perceived happiness level in a country, the more COVID-19 cases and deaths. Greater happiness is closely correlated with higher income, greater social support from relatives or friends, and greater freedom to make life choices^31^. Thus, higher levels of happiness may promote social interactions, leading to more infections and deaths. It is also plausible that people in happier countries may be more individualistic^32^, place a higher value on pleasure and freedom^33^, and resist restrictions and inconvenient prevention measures, such as wearing masks.

### Mitigating factors

#### Temperature

Prior studies (e.g.^34,35^) document that viral respiratory diseases present seasonal cyclicity, which can be driven by seasonal changes in temperature, sunlight, absolute humidity, vitamin status, and host behavior^36^. In line with these scientific findings, our results show that temperature negatively correlates with the severity of a coronavirus outbreak, suggesting that high temperatures may hamper the survival and spread of coronaviruses. This result is consistent with findings in recent studies on COVID-19^37,38^.

Note that the negative effects of temperature on confirmed cases and deaths do not imply that COVID-19 does not severely affect warm countries. Unlike SARS, which occurred during the winter months but disappeared abruptly as temperature increased, COVID-19 has hit several tropical countries hard, including India and Brazil. Thus, our findings only indicate that, all else being equal, average COVID-19 outcomes are less severe in warmer countries. Relatedly, Tushabe^39^ compares COVID-19 severity between tropical and temperate countries and documents that the average severity (in terms of infections or deaths) in temperate countries was six times as high as that in tropical countries.

#### Education

We find that a country’s education level, measured using the average number of years spent in school, is a significant factor that helps reduce confirmed cases and deaths. Using the coefficient of *education* in columns (1) and (3), we can infer that a one-standard-deviation (i.e., 2.772) increase in *education* is associated with a decrease of 14.41% (19.96%) in *confirmed* (*death*) from its mean value. This result is consistent with Cutler and Lleras-Muney^40^, who document a positive and persistent association between education and health, showing that different levels of education result in different thinking and decision-making patterns. In particular, better-educated people trust science more, are better informed, and exhibit higher levels of critical thinking and superior decision-making abilities. Relatedly, Zhong et al.^41^ conduct a knowledge, attitude, and practices survey after the coronavirus outbreak in China and find that most Chinese residents with relatively high socio-economic status are more informed about the COVID-19 situation and more proactive in prevention practices. Taken together, we believe that greater awareness and cognitive abilities associated with higher educational levels help explain the beneficial role of education in combating pandemics.

#### Religious diversity

Our measure of religious diversity (fractionalization) considers both the number and size of religions in a country. The results show that *religious diversity* is inversely associated with the number of infections or deaths. In additional analyses (untabulated), we examine the impact of ethnic and language diversity on the number of confirmed and death cases but find no significant results. Prior studies have documented that religion helps people cope with anxieties and trauma, develop resilience, and obtain social support from religious groups (e.g.^42^). Further, religion may also help discipline individuals and families, especially in countries where adherence to governments’ restrictive measures is weak.

More importantly, religious diversity reflects the differences in religious beliefs and practices in a country. Lu and Yang^43^ show that religious fractionalization is beneficial to self-rated health using survey data from 55 countries. They argue that diverse religious groups facilitate the accumulation of social capital toward one another, which benefits health and personal development. Alesina et al.^44^ document that religious fractionalization displays positive correlations with several measures of good governance across countries, and the correlation tends to be higher in more tolerant and free societies. Thus, our findings indicate that the coronavirus does not travel well across religious borders, and a tolerant and appreciative view of various religions helps curb the pandemic.

#### Media freedom

Prior studies document that the media has external governance functions in detecting corporate fraud^45^, exposing violations^46^, and monitoring corporate managers^47^. Our results reveal that the degree of media freedom is negatively correlated with COVID-19 cases, suggesting that a strong and free news media can help prevent the spread of infectious diseases. Timely and transparent information is critical for successful pandemic control. As many people live in confinement for months during a pandemic, they become increasingly reliant on various types of media for news and information to understand the COVID-19 crisis, protect themselves, and evaluate governments’ responses and policies. Media freedom can facilitate the dissemination of critical information about COVID-19 and stop the spread of fake news and misinformation so that citizens have more time to respond to COVID-19 and obtain sufficient, trustworthy information to make informed decisions. The media also fulfills the role of checking government policies and combating corruption. As such, freedom of expression and the press, instead of information control, is much needed in pandemics and should be highly valued as a core part of the social infrastructure required to beat COVID-19.

Interestingly, we find that media freedom is positively associated with the number of deaths. A plausible explanation is that countries with a free media are more truthful in counting and reporting COVID-19 deaths^48^. Using a machine-learning model to estimate the true death toll of the pandemic, *The Economist* discovers that official death tolls undercount the total number of fatalities in many parts of the world (https://www.economist.com/graphic-detail/coronavirus-excess-deaths-tracker). To gain reputational benefits and avoid scrutiny and criticism, a government can curtail media freedom, monopolize access to COVID-19 information, and suppress the true scale of deaths. A few countries (e.g., Egypt, Iran, and Hungary) have banned the dissemination of pandemic-related statistics except those released by officials. In its 2021 media freedom index, Reporters Without Borders reveals a “dramatic deterioration in people’s access to information and an increase in obstacles to news coverage,” suggesting that many governments have used the COVID-19 pandemic to prevent journalists from accessing information and to restrict critical reporting (https://theconversation.com/press-freedom-how-governments-are-using-covid-as-an-excuse-to-crack-down-on-the-publics-right-to-know-159298). In contrast, an independent press can help hold governments accountable and mitigate the underreporting of COVID-19 deaths^48^, implying a positive effect of media freedom on reported death cases, *ceteris paribus*.

#### Female leader

We find that, other things being equal, countries with women holding the highest political position generally perform better in the coronavirus pandemic than those with male leaders. Specifically, the coefficients of *female leader* in columns (1) and (3) imply that, on average, female-led countries have a 25.4% (38%) lower number of confirmed cases (deaths) than male-led countries.

This finding is generally consistent with Coscieme et al.^49^, who analyze data from 34 developed countries and China and conclude that female leaders lead to better COVID-19 control outcomes. Their study focuses on countries that have high economic, human development, and democracy indexes, with the exception of China, which was included since it was the first country to report COVID-19 cases. Coscieme et al.^49^ argue that both contingent factors, such as timeliness and decisiveness of intervention policies, and structural factors, such as higher equality and social development, may explain their findings. Conversely, Aldrich and Lotito^50^ empirically find no evidence that female leaders introduce containment measures and information campaigns earlier.

Using a much larger sample that contains 99 developed and developing countries, our findings confirm that leadership gender helps explain cross-country performance variations in the pandemic. During the COVID-19 pandemic, female leaders have generally acted more quickly and decisively and have demonstrated greater risk aversion toward losses of human life^51^. In addition, they have consistently taken a broader view to consider the wider impact of coronavirus on society and have been more open to innovative thinking^52^, thereby managing the COVID-19 crisis better than their male counterparts.

#### Trust government

We find that the proportion of people who trust the national government is inversely related to the number of confirmed cases and deaths. Higher levels of trust in the government boost conformance with government regulations. Siegrist and Zingg^53^ show that confidence in health agencies positively affects people’s willingness to adopt recommended behavior. Trust in public institutions is also vital for governments’ ability to respond rapidly and effectively and secure support during the pandemic. Chang et al.^14^ argue that trust in government and societal compliance can be more effective than authoritative lockdowns, as evidenced by Japan and South Korea, whose governments did not resort to large-scale lockdowns.

#### Law

*Law* measures the strength of a country’s legal system and the degree of observance of law and order. Countries that rank highly on this variable have a more law-abiding citizenry. As the vast majority of countries have adopted containment measures at least at certain points during their pandemic control, this variable reflects the degree to which the containment measures and other government interventions have been observed by the citizenry. The strength and impartiality of the legal system should directly drive the effectiveness of government interventions against COVID-19.

#### SARS

The negative and significant coefficients of *SARS* indicate that countries with SARS experience have achieved better performances in combating COVID-19. This finding is consistent with Ru et al.^54^, who find that countries with SARS experience have responded to the pandemic in a more timely and proactive way. Their findings are supported by Google search data and evidence from containment policies enforced by central governments. Using the imprint theory, they attribute their results to memories that people have of earlier SARS outbreaks and corresponding preparations. We expect that countries/regions hit by similar viruses, especially those with more recent experiences, may be more alert and responsive. For example, South Korea’s experience with Middle East Respiratory Syndrome in 2015 has also helped the country cope with COVID-19 outbreaks. In sum, our results imply that countries with a history of epidemics have clear advantages in preparing for new health catastrophes. Interestingly, Fig. 3 shows that countries/regions with SARS experience had more confirmed COVID-19 cases in the first seven weeks (ending on March 11, 2020) of our sample. One possible explanation is that China, as a country with SARS experience and the first country to report COVID-19 cases, reported a sharp increase in confirmed cases during our early sample period.

#### Hospital beds

The number of hospital beds is an important measure of healthcare infrastructure. Our results show that this is negatively associated with both infections and deaths, confirming that the overall healthcare infrastructure and medical resources in a country are vital for combating pandemics.

#### N_Tests

The number of people tested for COVID-19 is not a predetermined country characteristic, but it can be mechanically related to the number of infected cases reported. Thus, we include the number of cases tested as a control variable in columns (2) and (4) of Table 2. The results show that testing for coronavirus is positively related to the number of infections. This result is not surprising as the more tests conducted, the higher the discovery of confirmed cases. In other words, COVID-19 testing helps identify more infected people. In contrast, the number of cases tested is negatively related to the number of deaths, suggesting that more accurate and reliable tests significantly lower COVID-19 mortality by facilitating earlier detection, isolation, and treatment. More importantly, including the number of COVID-19 tests in regressions does not materially affect the coefficients of the predetermined country characteristics discussed above.

### Additional analyses

To examine the time-varying effects of our cross-sectional country characteristics on the severity of COVID-19 outcomes, we plot the weekly coefficients of each variable over time for confirmed cases (deaths) in Fig. 3a,b. The results show that these weekly coefficients vary over time. Specifically, the coefficients estimated during the early weeks of the pandemic are more volatile and less statistically significant than those in the later weeks. In addition, the coefficient signs are generally persistent across time. Two exceptions are *SARS* and *hospital beds,* which appear to aggravate confirmed cases and deaths during the first few weeks of our sample but become mitigating factors after week 8.

**Figure 4:**
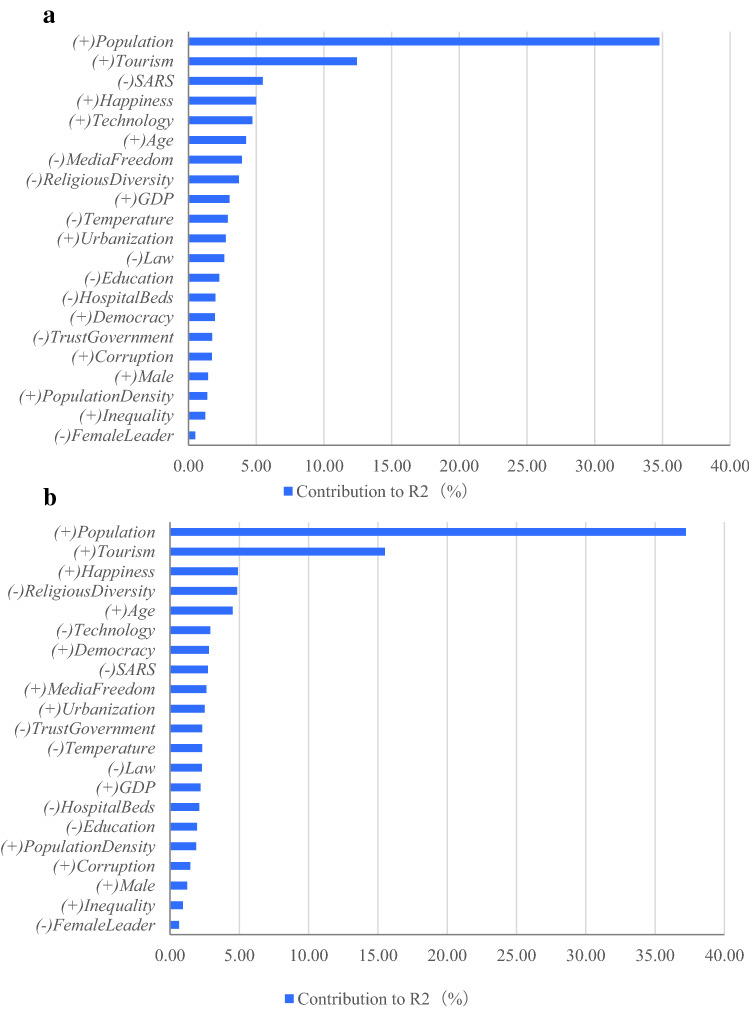
Dominance analysis. **(a)** Shows the Shapley–Owen R^2^ decomposition analysis for confirmed COVID-19 cases using 21 determinants as the independent variables. The figure ranks determinants based on their contributions to R^2^. The sum of the contributions is 100%. A positive (negative) sign indicates whether the determinant increases (decreases) confirmed COVID-19 cases. **(b)** Shows the Shapley–Owen R^2^ decomposition analysis on COVID-19 deaths using 21 determinants as the independent variables. The figure ranks determinants based on their contributions to R^2^. The sum of the contributions is 100%. A positive (negative) sign indicates whether the determinant increases (decreases) deaths.

To assess the relative statistical importance of the determinants in our regressions, we perform a Shapley–Owen R^2^ decomposition analysis and determine the dominance of one determinant over another by comparing their additional R^2^ contributions across all subset models. Figure 4a,b present the relative R^2^ contributions of all determinants in the confirmed cases and deaths regression, respectively. The relative R^2^ contributions are ranked in descending order. The results show that *population* is the most important determinant of confirmed COVID-19 cases (deaths); specifically, *Population* explains approximately 34.8% (37.2%) of cross-country variations in confirmed cases (deaths). The top five determinants (*population*, *tourism*, *SARS, happiness*, and *technology*) combined contribute about 62.5% of the R^2^ of the confirmed cases regression.

**Figure 5:**
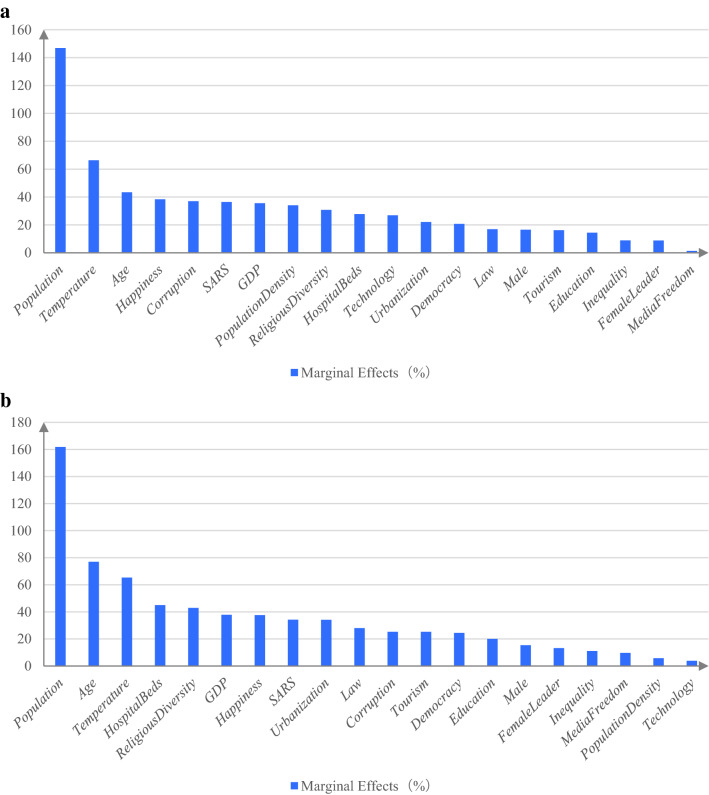
Marginal effects of a one-standard-deviation change in determinants. **(a)** Summarizes the marginal effects of a one-standard-deviation change for each of the determinants of confirmed COVID-19 cases. After varying each determinant by one standard deviation, the percentage change in the number of confirmed cases from the mean value is computed using the coefficients reported in Table 2. The marginal effects of all determinants are ranked in descending order based on their absolute values. **(b)** Summarizes the marginal effects of a one-standard-deviation change for all determinants of COVID-19 deaths. After varying each determinant by one standard deviation, the percentage change in the number of deaths from the mean value is computed using the coefficients reported in Table 2. The marginal effects of all determinants are ranked in descending order based on their absolute values.

Next, we summarize the marginal effects of a one-standard-deviation change for all determinants. Specifically, we vary each determinant by one standard deviation and compute the percentage change in the number of confirmed cases (deaths) from its mean value using the coefficients reported in Table 2. Figure 5a,b rank the marginal effects of all determinants in descending order based on their absolute values in the morbidity and mortality regressions, respectively. The results show that population, temperature, and age are the three determinants with the strongest marginal effects on the number of confirmed cases and deaths.

Finally, our analysis so far has primarily focused on the independent effects of predetermined country characteristics on COVID-19 outcomes. However, these characteristics may depend on one another in driving COVID-19 morbidity and mortality across countries. To examine this, we employ interaction terms among the country characteristics. However, since the possible two-way interaction terms among the 21 determinants are numerous, we use a machine-learning method, LASSO (least absolute shrinkage and selection operator), to identify and select potentially important interaction terms among all possible alternatives. Interestingly, Fig. 3 shows that countries/regions with SARS experience had more confirmed COVID-19 cases in the first 7 weeks (ending on March 11, 2020) of our sample. One possible explanation is that China, as a country with SARS experience and the first country to report COVID-19 cases, reported a sharp increase in confirmed cases during our early sample period. Our selection criterion is that selected interaction terms need to be statistically significant in 80% of weeks for both morbidity and mortality regressions. We find that *population* $\times$ *happiness* is the only interaction term meeting the selection criterion. We then add this interaction term to our baseline model and re-estimate the Fama and MacBeth regressions. The results (untabulated) show that the coefficients on *population* $\times$ *happiness* are significantly positive for both regressions, implying that the aggravating effect of happiness on COVID-19 outcomes is more pronounced in more populous countries.

## Discussion and conclusions

Our analysis of the relation between 21 predetermined, country-specific factors and COVID-19 outcomes suggests that multiple factors are required to explain country-level performances in the battle against COVID-19. Ex-post contingent interventions are certainly crucial; however, pre-existing country characteristics exogenous to the outbreak can explain a substantial part of pandemic outcomes.

To mitigate pandemic severity and risk, populous countries should remain on alert because most work and social activities occurs domestically, and population acts as a decisive aggravating factor for pandemic outbreaks. Since most countries are moving toward an aging population, it is crucial to protect the elderly, who are especially vulnerable during pandemics. As a result of urbanization, higher population density is unavoidable in many economies. Therefore, governments should strive to achieve a balance between the efficient utilization of urban spaces and sufficient social spaces for disease prevention in the layout design of housing and communities.

Both authoritarian and democratic governments must act in a unified and timely manner to manage health crises. For democratic countries, democracy itself is not to blame; rather, it is about whether leaders can strike a delicate balance between authority and liberal rights during the crisis. Policies should be put in place to allow governments to make centralized decisions in a health crisis, as delays in interventions can result in life and death consequences. In an outbreak, the life of every person is linked to the choices and behaviors of others; first and foremost, everyone has the right to secure their health. Thus, we believe that it is right and just for governments to develop awareness among the citizenry about the importance of sacrifices (e.g., loss of mobility) during critical periods of pandemic outbreaks. Governments and citizens should act in unity and prioritize pandemic control, even at the expense of reducing a certain degree of freedom in both political and individual domains. Factors (e.g., corruption) that give rise to inefficiencies in government, which, in turn, destroy trust in government, must be eradicated. Our findings suggest that democracy must be accompanied by a strong government for successful pandemic control. As Henderson^55^ observes, “as the pandemic has made clear, strong government—democratically accountable government, balanced by a free media and a thriving private sector—is the price we pay for strong societies”.

The fact that COVID-19 has affected some developed countries more than developing countries indicates that economic power does not imply immunity to a health crisis. It is not the financial resources of a country but the timeliness and effectiveness of interventions that primarily determine the success of pandemic controls. Countries would be wise to realize that it is not only GDP that matters but also how such resources are allocated, and the endeavor to invest more resources in public health and disease prevention in addition to advances in medicine. Income inequality within a country can cause health disparities, and governments should consider health as a public good, making preventive healthcare as widely accessible as possible. Our findings on the heightening effect of income inequality on COVID-19 severity suggest that public policies should direct special attention to protecting the poor, who are more vulnerable to pandemics. Countries that rely heavily on tourism inflows may need to prepare for increased health risks associated with the spread of infectious diseases, in addition to those risks related to the inevitable reductions in income resulting from the effects of the pandemic on travel.

To enhance the mitigating factors, investment in education has proved fruitful, even leading to an improvement in public health. Education enables citizens to make more informed health-related decisions. The control of a pandemic requires the participation and cooperation of every individual as citizens join governments to co-create social values. Countries should continue to develop the knowledge of citizens, especially as technologies (e.g., contact tracing) become instrumental for crisis management. Technological innovation can facilitate infectious disease control and ensure that infected people are identified, traced, and treated in a timely manner, all of which can alleviate severe consequences in pandemics.

It is unrealistic to expect all countries to choose female leaders. However, perhaps male leaders could learn from their female counterparts and pay more attention to issues that matter to the health of the broader population and society. Trust in government, law, and order, which take a long time to develop, build a country’s resilience and have proved instrumental during both peace and crises. Past experience of similar health crises helps governments and citizens stay alert and better prepared for new health crises, as seen in countries with a history of SARS or similar. Investment in healthcare facilities pays off when a crisis escalates, but is justifiable with or without a crisis.

To conclude, we identify a set of predetermined country characteristics that significantly influence the outcomes of COVID-19. Country-specific factors can either strengthen or attenuate a country’s resilience to the pandemic. Our results have implications for policymakers, healthcare experts, and the public. Policymakers and healthcare experts may consider utilizing these factors to further develop risk-preparedness and strengthen social solidarity in health crises.

Despite its contributions, this study has at least two major limitations. The first relates to its reliance on *reported* infection cases and deaths. Reported figures may vary significantly across countries in terms of timeliness, accuracy, completeness, and consistency. For example, Belgium, a developed country, has aggressively reported COVID deaths that occurred in elderly-care homes based on symptoms and contacts, even without confirmed diagnosis. In contrast, some developing or authoritarian countries may have severely underreported the number of fatalities. Further, in countries with limited testing capacities, only the most seriously ill people can be tested. As such, many infected individuals who were asymptomatic may not have been included in our dataset. Such inequality in data quality and potential underreporting issues are a challenging—but potentially fruitful—avenue for future studies. Second, our study mainly focuses on the cross-sectional differences in COVID-19 outcomes at a given point in time. Thus, our analysis cannot reveal how and whether certain factors influence the transmission dynamics across countries, which would require time series models to explore the lead–lag relation among countries in confirmed cases and deaths. We leave this important issue for future research.

## Supplementary Information

Supplementary Information.
