# Supplementary material for: The determinants of COVID-19 morbidity and mortality across countries
Source: Sci Rep. 2022 Apr 7;12:5888. doi: 10.1038/s41598-022-09783-9 (PMC8989117; doi:10.1038/s41598-022-09783-9)
Supplement: Supplementary file 1 — Supplementary Information. [file 41598_2022_9783_MOESM1_ESM.pdf]

Supplementary Information (SI)

The Determinants of COVID-19 Morbidity and Mortality Across Countries

Dianna Chang[1]
[1]Singapore University of Social Sciences

Xin Chang[2]
[2]Nanyang Technological University

Yu He[3]
[3]Central University of Finance and Economics

Kelvin Jui Keng Tan[4,*]
[4]The University of Queensland

SI A. Variable Definitions

This appendix provides the names, definitions, and data sources of the variables used in the regressions.

Variable	Definition and Data Sources
Dependent Variables: COVID-19 Outcomes	
Ln(1+*Confirmed*)	The natural logarithm of one plus confirmed COVID-19 cases at the end of a week. Source: https://coronavirus.jhu.edu/map.html
Ln(1+*Death*)	The natural logarithm of one plus the number of COVID-19 deaths at the end of a week. Source: https://coronavirus.jhu.edu/map.html
Independent Variables: Demographic-Geographic Factors	
Population	The natural logarithm of a country's population in thousands in 2018. Source: World Bank
Population Density	The number of inhabitants (thousands of people) per square kilometer in 2019. Source: United Nations Population Division
Age	The median age of a country's population in 2018. Source: https://worldpopulationreview.com/
Male	The number of males per 100 females in the population in 2020. Source: https://population.un.org/wpp/Download/Standard/Population/
Urbanization	The proportion of people living in urban areas, as defined by national statistical offices, in relation to the total population. The data are collected and smoothed by the United Nations Population Division. Source: https://data.worldbank.org/indicator/SP.URB.TOTL.IN.ZS
Temperature	The weekly average temperature in Celsius from January to the end of every week. Source: World Bank
Education	The average number of years spent in school for a country's population. Source: https://ourworldindata.org/global-education
Religious Diversity	An index calculated based on the shares of eight major world religions (Buddhism, Christianity, folk religions, Hinduism, Judaism, Islam, other religions considered as a group, and the religiously unaffiliated). The index is on a scale from 0 to 10, with 10 being the maximum possible level of diversity if each of the eight groups constitutes an equal share of the population. Source: Pew Research Center
Independent Variables: Political-Legal Factors	
Democracy	A measure of whether there are free and fair elections in a country and how responsive a government is to its people. The higher the value, the more responsive the government. Source: International Country Risk Guide Dataset (ICRG)
Corruption	A measure of how corrupt a political system and a business environment are. The measure covers various forms of corruption, including excessive patronage, nepotism, secret party funding, and bribery. The higher the value, the more corrupt a country. Source: ICRG
Media Freedom	A measure that captures the degree of freedom available to journalists in a country. It is constructed by pooling the responses of experts to a questionnaire devised by Reporters Without Borders (RSF). The questionnaire-based qualitative analysis is combined with quantitative data on abuses and acts of violence against journalists during the period evaluated. The criteria evaluated in the questionnaire are pluralism, media independence, media environment and self-censorship, legislative framework, transparency, and the quality of the infrastructure that supports the production of news and information We multiply the RSF index by −1, so that a higher value implies a higher degree of media freedom. Source: https://rsf.org/en/ranking
Female Leader	A country leader is defined as the person holding the highest political position in a country; for example, the head of a state (i.e., president) or

Variable	Definition and Data Sources
	government (prime minister). *Female Leader* takes a value of 1 if the leader in a country is female and 0 otherwise. Source: We manually collect the gender of leaders from governments' official websites
Trust Government	The proportion of people who trust their national government. The Wellcome Global Monitor surveys over 140,000 people from more than 140 countries. Among other questions, survey respondents are asked "How much do you trust your national government? Do you trust them a lot, some, not much, or not at all?" *Trust Government* is computed as the fraction of people whose responses are "a lot" or "some." Source: https://wellcome.ac.uk/reports/wellcome-global-monitor/2018
Law	A measure that consists of two elements – law and order – assessed separately. Each element is scored from zero to three points. To assess the "law" element, the strength and impartiality of the legal system are considered. The "order" element is an assessment of popular observance of the law. A high score indicates a strong judicial system, but a low rating implies that the law is routinely ignored without effective sanctions. Source: ICRG
Independent Variables: Socio-Economic Factors	
GDP	GDP per capita in thousands, adjusted by purchasing power parity (PPP).
Inequality	The income/wealth distribution across a nation's population measured by the Gini coefficient. A higher Gini coefficient implies greater inequality, with those earning high incomes obtaining much larger percentages of the total income of the population. Source: World Bank
Tourism	The number of international tourist arrivals in a year. Source: World Bank
Technology	This variable measures the level of technological innovation related to digital and mobile networks and social media. It covers investment in emerging technology, 4G mobile network coverage, and the use of virtual social networks. We use the average value of these three technology aspects to capture the level of technological innovation. Source: World Economic Forum, the International Telecommunication Union database, Global Digital Report 2020
Happiness	The measure of life satisfaction (2017–2019). Source: World Happiness Report 2020
Independent Variables: Healthcare Factors	
SARS	*Ln*(1+*Confirmed Case*), where confirmed cases are caused by SARS. Source: https://www.who.int/csr/sars/country/table2004_04_21/en/
Hospital Beds	Hospital beds (per 1,000 people). Source: World Bank
Control Variable	
N_Test	The number of people tested for COVID-19 (per 100 people). Source: https://www.worldometers.info/coronavirus/

SI B. Countries and Regions Included in the Sample

This table shows the list of countries and regions in our sample. The number of confirmed cases of and deaths resulting from COVID-19 are taken from the Coronavirus Resource Center of Johns Hopkins University at the end of 2020. The six countries/regions with missing values for *Trust Government* are China, Egypt, Hong Kong SAR of China, Jamaica, Saudi Arabia, and Trinidad. The two countries with missing values for *N_Test* are Algeria and Tanzania.

Countries	Confirmed	Death	Countries	Confirmed	Death
**Africa: 18 countries**			**Europe: 34 countries**		
Algeria	99,610	2,756	France	2,677,666	64,759
Botswana	14,805	42	Germany	1,760,520	33,791
Cameroon	26,277	448	Greece	138,850	4,838
Egypt, Arab Rep.	138,062	7,631	Hungary	322,514	9,537
Ethiopia	124,264	1,923	Iceland	5,754	29
Kenya	96,458	1,670	Ireland	91,779	2,237
Madagascar	17,714	261	Italy	2,107,166	74,159
Malawi	6,583	189	Latvia	40,904	635
Mali	7,090	269	Lithuania	140,579	1,458
Morocco	439,193	7,388	Luxembourg	46,415	495
Mozambique	18,642	166	Malta	12,774	219
Nigeria	87,607	1,289	Moldova	144,818	2,985
Senegal	19,140	410	Netherlands	808,382	11,525
Tanzania	509	21	Norway	49,567	436
Tunisia	139,140	4,676	Poland	1,294,878	28,554
Uganda	35,216	251	Portugal	413,678	6,906
Zambia	20,725	388	Romania	632,263	15,767
Zimbabwe	13,867	363	Russian Federation	3,127,347	56,271
**Asia: 24 countries/regions**			Sweden	437,379	Serbia
Armenia	159,409	2,823	Slovak Republic	179,543	2,138
Azerbaijan	218,700	2,641	Slovenia	122,152	2,697
Bangladesh	513,510	7,559	Spain	1,928,265	50,837
China	87,071	4,634	Sweden	437,379	8,727
Cyprus	22,019	119	Switzerland	452,296	7,645
Hong Kong SAR, China	8,846	148	Ukraine	1,086,997	19,281
India	10,266,674	148,738	United Kingdom	2,496,231	73,622
Indonesia	743,198	22,138	**North America: 10 countries**		
Iran, Islamic Rep.	1,225,142	55,223	Canada	584,409	15,632
Israel	423,262	3,325	Dominican Republic	170,785	2,414
Japan	235,811	3,292	El Salvador	45,960	1,327
Jordan	294,494	3,834	Guatemala	138,012	4,813
Kazakhstan	201,196	2,761	Honduras	121,827	3,130
Korea, Rep.	61,769	917	Jamaica	12,827	302
Lebanon	181,503	1,468	Mexico	1,426,094	125,807
Malaysia	113,010	471	Panama	246,790	4,022
Mongolia	1,220	1	Trinidad and Tobago	7,150	127

Countries	Confirmed	Death	Countries	Confirmed	Death
Pakistan	482,178	10,176	United States	19,968,088	345,737
Philippines	474,064	9,244	**Oceania: 3 countries**		
Saudi Arabia	362,741	6,223	Australia	28,425	909
Singapore	58,599	29	New Zealand	2,162	25
Sri Lanka	43,299	204	Guinea	13,722	81
Thailand	7,163	63	**South America: 10 countries**		
Turkey	2,208,652	20,881	Argentina	1,625,514	43,245
**Europe: 34 countries**			Bolivia	160,124	9,165
Albania	58,316	1,181	Brazil	7,675,973	194,949
Austria	360,815	6,222	Chile	608,973	16,608
Belgium	646,496	19,528	Colombia	1,642,775	43,213
Bulgaria	202,266	7,576	Ecuador	212,512	14,034
Croatia	210,837	3,920	Paraguay	107,932	2,262
Denmark	164,116	1,298	Peru	1,015,137	37,680
Estonia	27,990	229	Uruguay	19,119	181
Finland	36,107	561	Venezuela, RB	113,558	1,028

SI C. Robustness Checks: Confirmed Cases and Deaths Scaled by Population

This table presents the results of robustness tests using the alternative measures of confirmed cases and deaths. The dependent variable in columns (1) and (2) is the natural logarithm of one plus confirmed COVID cases per million people, while the dependent variable in columns (3) and (4) is the natural logarithm of one plus the number of deaths per million people. The definitions of all variables are included in SI A. Columns (2) and (4) further control for the number of COVID-19 tests conducted (*N_Test*) and *Trust Government*. The *t*-statistics are reported in parentheses. ***, **, and * indicate significance at the 1%, 5%, and 10% levels, respectively.

	(1)	(2)	(3)	(4)
	***Ln*(1+*Confirmed*)**		***Ln*(1+*Death*)**	
Population	0.055***	0.084***	0.217***	0.227***
	(2.9)	(3.9)	(10.3)	(10.3)
Population Density	0.370***	0.367***	0.125***	0.094***
	(10.5)	(13.5)	(5.9)	(6.2)
Age	0.043***	0.047***	0.068***	0.062***
	(11.4)	(11.6)	(16.1)	(16.5)
Male	0.025***	0.019***	0.018***	0.024***
	(13.7)	(6.5)	(11.7)	(9.9)
Urbanization	0.010***	0.010***	0.011***	0.010***
	(20.3)	(16.2)	(12.9)	(10.9)
Temperature	-0.070***	-0.067***	-0.066***	-0.071***
	(-11.2)	(-11.7)	(-12.2)	(-12.4)
Education	-0.040***	-0.063***	-0.043***	-0.057***
	(-11.7)	(-10.0)	(-10.9)	(-9.4)
Religious Diversity	-0.143***	-0.131***	-0.165***	-0.155***
	(-12.4)	(-13.1)	(-10.2)	(-10.2)
Democracy	0.162***	0.125***	0.143***	0.131***
	(11.5)	(9.5)	(13.1)	(12.5)
Corruption	0.258***	0.235***	0.210***	0.177***
	(19.4)	(18.1)	(15.1)	(15.7)
Media Freedom	-0.000	-0.003**	0.011***	0.008***
	(-0.5)	(-2.6)	(9.7)	(5.3)
Female Leader	-0.271***	-0.127***	-0.395***	-0.379***
	(-6.4)	(-5.2)	(-10.2)	(-11.7)
Trust Government		-0.006***		-0.007***
		(-6.1)		(-7.4)
Law	-0.149***	-0.037*	-0.153***	-0.111***
	(-4.7)	(-1.7)	(-8.5)	(-7.7)
GDP	0.016***	0.014***	0.015***	0.017***
	(15.9)	(14.9)	(11.7)	(13.8)
Inequality	0.016***	0.019***	0.021***	0.019***
	(6.3)	(9.9)	(9.4)	(11.0)
Tourism	0.008***	0.006***	0.012***	0.011***
	(9.0)	(5.2)	(10.3)	(8.4)
Technology	0.010***	0.008***	-0.004***	-0.004***
	(7.5)	(5.4)	(-4.7)	(-3.4)
Happiness	0.350***	0.317***	0.349***	0.359***
	(9.6)	(8.3)	(9.5)	(9.3)
SARS	-0.318***	-0.273***	-0.290***	-0.321***
	(-8.3)	(-11.8)	(-11.8)	(-14.5)
Hospital Beds	-0.126***	-0.134***	-0.180***	-0.185***
	(-14.4)	(-14.6)	(-15.8)	(-16.0)
N_Tests		0.002***		-0.001***
		(6.1)		(-4.6)
Constant	0.459	0.918	-3.112***	-3.247***

	(1)	(2)	(3)	(4)
	***Ln*(1+*Confirmed*)**		***Ln*(1+*Death*)**	
	(1.5)	(1.6)	(-11.6)	(-10.7)
N	4851	4459	4851	4459
R^2	0.686	0.679	0.640	0.602

SI.D. Regarding the maps in Figures 1a and 1b, we used Stata 15.1 to generate the maps. More specifically, we calculated the confirmed/dead cases based on the data from https://www.worldometers.info/coronavirus/. We obtained the world map data from http://fmwww.bc.edu/repec/bocode/w/. We downloaded world-c.dta and world-d.dta, to get information about countries' location, area, perimeter, country-code, etc. We then used the code of "spmap" in Stata to generate the map.
